# In Response

**DOI:** 10.4269/ajtmh.2011.10-0587b

**Published:** 2011-01-05

**Authors:** Remington L. Nevin, Lénaïck Ollivier

**Affiliations:** Bayne-Jones Army Community Hospital; Fort Polk, LA; E-mail: remington.nevin@us.army.mil; Direction Centrale du Service de Santé des Armées; Paris, France; E-mail: lenaickollivier@hotmail.com

Dear Sir:

We thank Dr. Coton for his kind letter^1^ and agree with his assessment that other gastrointestinal pathogens not specifically addressed in our recent paper, particularly *Giardia duodenalis*, present ongoing challenges to military personnel serving on deployments in Djibouti, and elsewhere.

Although published information is lacking on the incidence of giardiasis specific to deployed French Forces, a research study conducted between 1992 and 1993 during a previous United States military operation in neighboring Somalia found *Giardia* in 3 of 113 specimens (3%) collected from service members presenting with diarrhea or with high fever.^2^ These personnel had limited exposure to local food and water sources; the risk posed by outbreaks of giardiasis among those for whom such exposures are intrinsic aspects of duty would likely be much higher than suggested by this report.

For cases of persistent diarrhea thought caused by *Giardia* infection, presumptive treatment with either metronidazole or albendazole are both equally effective and each are fairly well tolerated among adults.^3^

Despite the availability of such therapies, improving opportunities for definitive diagnosis of *Giardia* infection should remain a priority. The wider use of rapid assays for diagnosis may help to overcome many of the limitations found in certain deployed settings where adequate laboratory facilities are often lacking, and thus aid in improving surveillance in settings where this infection may be a significant and unappreciated cause of prolonged gastrointestinal illness.
